# Associations of women's position in the household and food insecurity with family planning use in Nepal

**DOI:** 10.1371/journal.pone.0176127

**Published:** 2017-04-28

**Authors:** Nadia Diamond-Smith, Anita Raj, Ndola Prata, Sheri D. Weiser

**Affiliations:** 1 University of California San Francisco, San Francisco, California, United States of America; 2 University of California San Diego, San Diego, California, United States of America; 3 University of California, Berkeley, California, United States of America; Western Sydney University, AUSTRALIA

## Abstract

**Background:**

Women in Nepal have low status, especially younger women in co-resident households. Nepal also faces high levels of household food insecurity and malnutrition, and stagnation in uptake of modern family planning methods.

**Objective:**

This study aims to understand if household structure and food insecurity interact to influence family planning use in Nepal.

**Methods:**

Using data on married, non-pregnant women aged 15–49 with at least one child from the Nepal 2011 Demographic and Health Survey (N = 7,460), we explore the relationship between women’s position in the household, food insecurity as a moderator, and family planning use, using multi-variable logistic regressions. We adjust for household and individual factors, including other status-related variables.

**Results:**

In adjusted models, living in a food insecure household and co-residing with in-laws either with no other daughter-in-laws or as the eldest or youngest daughter-in-law (compared to not-co-residing with in-laws) are all associated with lower odds of family planning use. In the interaction model, younger-sisters-in-law and women co-residing with no sisters-in-law in food insecure households have the lowest odds of family planning use.

**Conclusion:**

This study shows that household position is associated with family planning use in Nepal, and that food insecurity modifies these associations–highlighting the importance of considering both factors in understanding reproductive health care use in Nepal. Policies and programs should focus on the multiple pathways through which food insecurity impacts women’s reproductive health, including focusing on women with the lowest status in households.

## Introduction

Family planning is a key factor in improving both women and children’s health and socio-economic status. The percent of women globally who want to avoid pregnancy and are using family planning increased slightly from 54% to 57% between 2003 and 2008, but did not increase further by 2012 [1]. South Asia, while having higher rates of family planning use than the global average, followed this same trend, with this percent increasing from 61% to 66% between 2003 and 2008, and also stagnating at 66% by 2012 [1]. The 2011 Nepal Demographic and Health Survey (DHS) found a small decrease in the use of modern family planning methods (from 44% in 2006 to 43% in 2011), although overall family planning use (modern and traditional) increased slightly (from 48% in 2006 to 50% in 2011) [2]. Modern methods of family planning include the oral contraceptive pill, female and male sterilization, IUD, injectables, implants, male and female condom, diaphragm, and emergency contraception [3]. Research has suggested that high rates of spousal separation, increased use of abortion, emergency contraception, and traditional methods, and also lack of focus on hard to reach and vulnerable populations for services, could be contributing to the lack of continued increase in modern method uptake in Nepal [2, 4]. Qualitative research in Nepal found that misinformation and fears of side effects were common barriers to modern family planning use, and family members, such as mothers-in-law and husbands, were important decision-makers about use of family planning [5]. Additionally, more communication between husbands and wives has been found to be associated with greater use of family planning in Nepal, as in other countries [6].

Women’s status relative to men in South Asia, including Nepal, is low compared to other parts of the world, as evidenced by large gaps between male and female literacy, vaccination rates, and weight-for-age scores, to name a few indicators [7]. Women’s status is associated with her health care use in Nepal, as elsewhere. In Nepal, women who had higher decision-making autonomy and better communication with husbands had increased use of antenatal care and delivery with a skilled provider and were less likely to experience violence [8–10]. Although there is a paucity of research on women’s status or autonomy in Nepal, most evidence suggests that there is geographic heterogeneity, with women in the Terai region generally being the least empowered [11]. One important factor influencing women’s status in Nepal is her position in the hierarchy of the household in which she lives. Three quarters of women in Nepal reported living in joint or co-residing households (households with other family members, usually the husband’s parents) [12]. These households can include not just a mother and father-in-law, but also other brothers and their wives, creating an extended family of multiple families within one household. Status within the household is traditionally based on the son’s birth order, with the eldest sons (and their wives) having the highest status [13]. When a young woman moves into her husband’s home in South Asia, social convention supports her coming under the control of her mother-in-law, and also elder sisters-in-laws [14, 15]. Past research in India found that women living in co-residing households were less likely to use antenatal care or deliver in a facility than women living in nuclear households [16]. Qualitative research in Nepal has found that mothers-in-laws are important decision makers about antenatal care use and family planning, highlighting the importance of understanding women’s exposure to her mother-in-law through co-residence in order to fully understand her health care use and outcomes [5, 17]. Due to a strong cultural preference for sons, one way that a young wife increases her status is through bearing a son, although any childbearing is associated with improved status [14].

Past qualitative work has suggested that women of low status in the household have access to fewer household resources, including food. Cultural beliefs dictating that women reduce food consumption while menstruating, pregnant, or lactating, and a general practice of young girls and new daughter-in-laws (i.e., women with lower status in households) eating last, lead women in Nepal to have unequal access (relative to men) to micronutrient rich foods in the household [18–20]. Other, mostly qualitative, research has suggested that the distribution of food in the household is based on household position, with young daughters-in-laws, especially those married to younger sons, often being the last to eat [20]. Past analysis of the 2011 Demographic and Health Survey (DHS) in Nepal found that factors associated with women’s status, including woman’s education, sex of household head, and urban/rural residence of households were associated with her anemia level, an indicator of poor health, however, they did not include a marker of household position [21].

Nepal suffers from high levels of food insecurity, with more than half of districts being found to have some level of food insecurity in the latest Demographic and Health Survey (2011) [22]. The severity of food insecurity differs between the different regions (mountains, Terai (plains near Indian boarder), hills) of Nepal, with households in the Terai being more food secure [23]. Food insecurity has been found to be associated with a broad range of poor health outcomes, including HIV, chronic disease, and mortality [24]. Food insecurity has also been found to be associated with low rates of exclusive breastfeeding and poor antenatal care seeking practices [25, 26]. Additionally, food insecurity has been found to be associated with increased sexual risk-taking behavior (for example, inconsistent condom use and sex exchange) and sexually transmitted disease symptoms, among women in Nepal, and elsewhere [27, 28]. We propose that food insecurity may impact family planning use both because of impacts on mental health and on empowerment. Food insecurity is a well-known predictor of depression, anxiety and poor mental health, which in turn may make it harder for women to seek services or advocate for the services that they want to use within their household or community [29, 30]. Food insecurity has also been associated with disempowerment in other studies and disempowerment in turn could impede family planning use. Even women who do not suffer from poor mental health due to food insecurity might find it difficult to seek or advocate for family planning services, which could be further compounded by being of low household status [31].

Food insecurity is difficult to measure, and different studies use a variety of approaches (sets of questions, inclusion of different components of food security such as access, utilization, availability, etc.) [32]. One primary concern with commonly used measures, such as the one used in the Nepal DHS, which is adapted (to be culturally relevant) from the Household Food Insecurity Access Scale indicators developed in USAID’s Food and Nutrition Technical Assistance (FANTA) project [33], is that it asks about household level food insecurity, and an individual’s experience of food insecurity within a household could be different from the whole household’s experience. The food insecurity questions are answered by the household head, which is usually a male in this setting and may reflect the heads experience, or his view of the household’s experience. Thus, much of the rational for this analysis is to try to understand how incorporating information about a woman’s status in the household helps us better understand what the household-level food insecurity measure (generally answered by a male household head) means for an individual woman within that household, hence addressing one of the main limitations of this measure.

**Fig 1:**
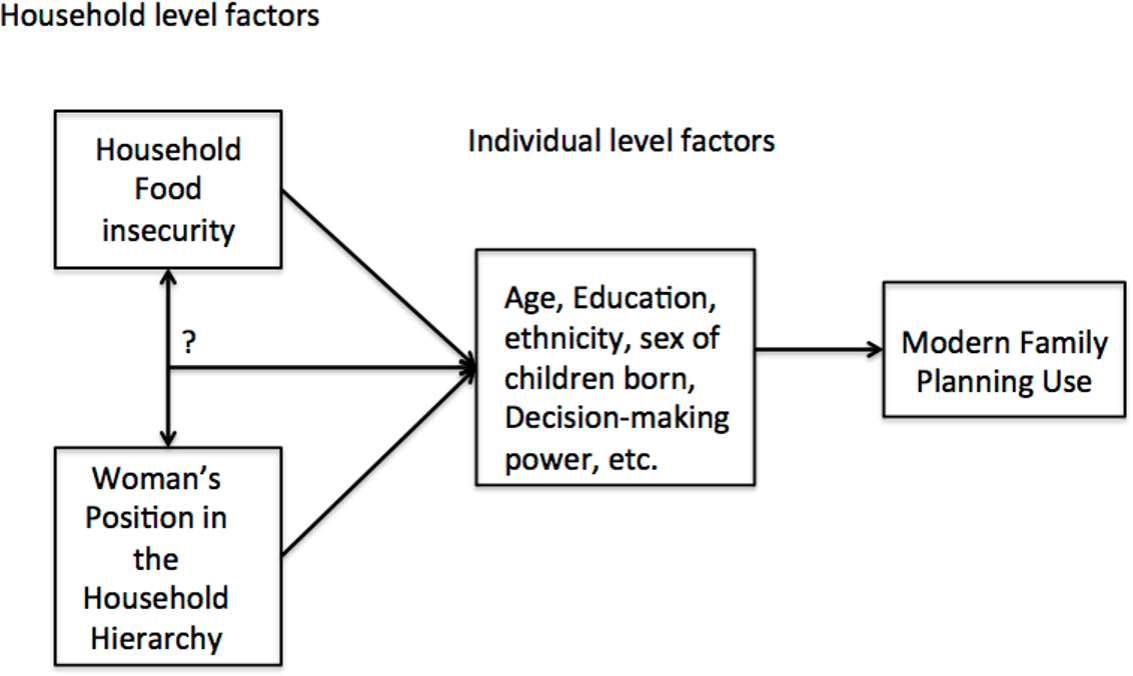
Conceptual and analytical model.

Although it is well recognized that women’s position in the household impacts her status, as described above, little research has explored the interconnection between household position, food insecurity, and reproductive health indicators, such as use of family planning. Furthermore, the little research that exists on the interrelationship between household status and health outcomes in this setting is relatively old, and much of it not nationally representative. We hypothesize that household position and food insecurity will be associated with modern family planning use, and that high food insecurity and low household position could interact to reduce family planning use. We hypothesize this because lower status women are both less likely to be able to access food in food insecure households, being food insecure further disempowers women, and thus women with both risk factors might be less likely to use modern family planning methods (Fig 1). Women might also be at low status in her household due to her age, education, if she is the household head alone, level of decision-making power, or how many/what sex of children she has. Additionally, household factors such as wealth, ethnicity, religion, region or urban/rural status, might put the household at further risk of being food insecure or have inequitable gender norms. The goal of this analysis is to first understand if women’s position in the household is associated with her use of modern family planning. Second, we explore whether food insecurity is associated with modern family planning use. Finally, we explore whether there is an interaction between woman’s position in the household, food insecurity, and women’s modern family planning use.

## Materials and methods

We use data from the 2011 Nepal Demographic and Health Survey (DHS) for this analysis. The DHS was collected under the guidance of Population Division, Ministry of Health and Population of the Government of Nepal and with technical assistance from ICF International. The DHS is a nationally representative household survey of women of reproductive age, defined as 15–49 years of age, conducted every 5 years using a stratified, two stage cluster design with a 97.6% response rate in 2011 [28]. It has household level as well as individual level modules for men and women. For this analysis we link the household level and individual women’s modules. The household module (which includes the household food insecurity questions) was answered by the household head and the individual women’s module was answered by selected women of reproductive age in that household. A total of 12,674 women were interviewed in 2011. For this analysis, the sample is limited to currently married, non-pregnant women with at least one living child. Since one of the main variables of interest is household structure, we focus on married women who are living in different types of co-resident structures. Non-married women would likely have very different household structures and be subjected to different roles in their households. We limit the sample to non-pregnant women since family planning use is not relevant among pregnant women. Additionally, different norms are likely to apply to pregnant women’s access to food. We also restrict the sample to women who had at least one living child, since family planning use before the first child is born is very low in this setting. A total of 7,459 women have no missing data and fit these criteria.

**Fig 2:**
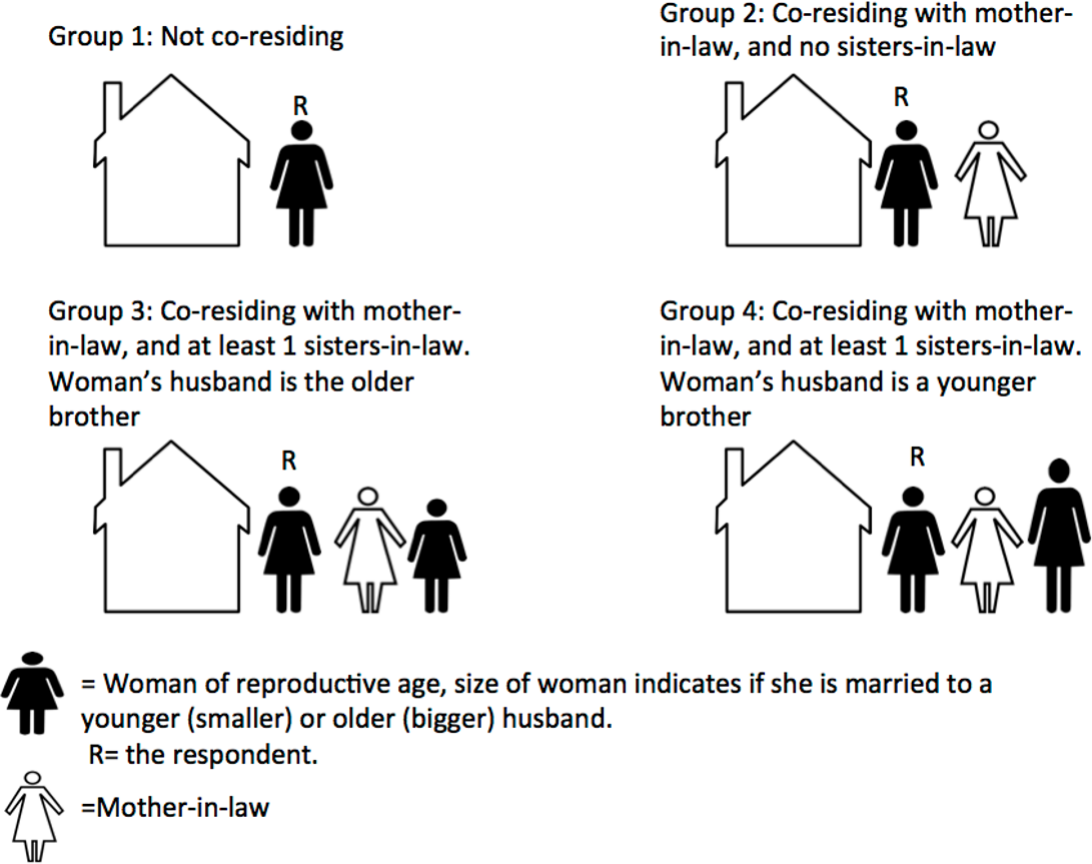
Categories of women’s position in the household.

Two primary factors potentially associated with the outcome of interest (family planning use) are explored in this analysis. The first is the household co-residence structure in which the woman lived. This variable is created by grouping women by household ID in the Nepal DHS data, since more than one woman of reproductive age per household was eligible to participate in the survey. Then, based on whether or not more than one woman in the household was included in the sample, and combined with information from the question “What is your relationship to the household head?” we categorize women into the following four groups: Group 1 includes women who were not co-residing, defined as women who reported that they were married to the head of the household or were the head of the household and were not living with another woman of reproductive age in the household, nor with a mother-in law. These women are the referent group as we assume them to be the highest status group. Group 2 includes woman living in a household with in-laws, but no sister-in-laws who took the survey. Group 3 includes women living in household where there was at least one other woman in the same household, and their husband was older than the other woman’s husband. This is the “elder” sister category. Group 4 includes women living with at least one other woman from the survey in the same household, and the husband being younger than the husband of the other woman in the household. If more than two women were included in the survey who lived in the same household, then the one with the oldest husband was in group three and all of the younger ones in group 4 (Fig 2). We hypothesize that Group 4 women have the lowest status in the household, and Group 1 the highest, however, it is unclear whether elder (Group 3) women or women with no sisters-in-law (Group 2) would be of higher status.

The second factor of interest is household level food insecurity. Food insecurity was measured using the household food insecurity access scale collected in the 2011 Nepal DHS [28]. The 2011 Nepal DHS asked 7 of the 9 questions in Household Food Insecurity Access Scale indicators developed in USAID’s Food and Nutrition Technical Assistance (FANTA) project [33]. These questions were modified to be culturally relevant to Nepal and the time frame was expanded to 12 months (from the standard 1 month) because of seasonal variability in the Nepali setting. Questions were answered by the head of the household (generally a male in this setting) and asked about insufficient quantity and quality of foods and anxiety and uncertainty about food supply. Compared to other measures, this set of questions does not address either individual household members access or more distal factors such as livelihood production [32]. We use a validated scoring algorithm, to create a four-group categorical variable ranging from “food secure” (1) to “severe food insecurity”, as is categorized by the Nepal DHS (4). For the interaction model, we created a binary variable, collapsing none/mild/moderate vs. severe food insecurity.

The primary outcome of interest is a binary for current use of a modern family planning method compared to no method or a non-modern method.

We use multivariate logistic regressions to assess for associations between food insecurity, women’s household status, and modern family planning use. We control for socio-demographic factors found in past literature to be associated with women’s household status, food insecurity and/or family planning use in this setting. At the household level, these include wealth quintile (calculated by the DHS Program, provided in the dataset), urban/rural status, geographic region (mountains, hills or Terai), caste/ethnicity (Brahmin/Chhetri, Newar, Dalit, Janajati, other), and religion (Hindu compared to any other religious group). At the individual level we control for women’s age (in 10 year age groups), education (none, primary, secondary, and higher than secondary, as categorized by the DHS Nepal), type of occupation (professional occupation vs. not), if she is the head of the household (yes/no), and the total number of living children that the woman has (continuous). Furthermore, we include a variable of the ratio of the number of boys divided by the number of girls that a woman has at the time of the survey, to account for the effect of having a son on a woman’s position in the household and probability of using modern family planning. We also include a score of women’s decision making level in the household. This is comprised of six binary questions about who (the woman alone, woman and her husband, husband alone, other) makes decisions about the following: what to do with money that the husband earns, what to do with the money the respondent earns, using family planning, the respondents health care, large purchases, and visits to family/relatives. These were then summed to create the final score (ranging from 0–6).

Since more than one woman could live in each household and the main predictor is a household level variable, each woman received a weight based on the number of women in the sample in her household (0.5 if there were two women, 0.33 if there were 3 women, etc.). This weight is multiplied by the survey weight provided by ICF Macro. Data were analyzed using STATA 12.

## Results

### Description of population

The mean age of the sample is 32.8 years. Most women (72.6%) live in rural areas, almost 45% (44.63) lived in the Terai, followed by hills (38.96) and 20.6% indicate that they are the household head. Approximately half (50.4%) of respondents have no schooling and very few (3.5%) have professional occupations. The mean decision-making score is 1.27 (out of a possible score of 6 representing the most decision-making power). Women have, as a median, 2.76 living children, with a ratio of 1.19 boys/girls. About half (49.9%) are currently using a modern family planning method. The most commonly used modern method is female sterilization (12.62%), followed by injection (10.19%) and male sterilization (8.67%).

Most women are not co-residing (63.6%). Over thirteen percent (13.7%) of women are co-residing in households without other sisters-in-laws, 14.0% co-residing in household where they were married to the eldest son and hence were the eldest sister-in-law, and 8.7% co-residing in households where they were a younger sister-in-law (had at least 1 older sister-in-law). Almost fifty percent (48.8%) of households are food secure, 13.5% mildly food insecure, 23.8% moderately food insecure and 14.0% severely food insecure (Table 1).

**Table 1:** Respondent characteristics ^#^N = 8,068.

	Number of respondents (%) / Median (Inter Quartile Range)
**Position in the household**	
Not co-residing	5,128 (63.56)
Co-residing, no other sisters-in-laws	1,107 (13.72)
Co-residing, married to eldest son	1,128 (13.98)
Co-residing, married to younger son	705 (8.74)
**Food Insecurity**	
None	3,933 (48.75)
Mild	1,086 (13.46)
Moderate	1,916 (23.75)
Severe	1,133 (14.04)
**Women’s Age**	32.77 (15–49)
**Urban (vs. rural)**	2,215 (27.45)
**Geographic Region**	
Mountain	1,324 (16.41)
Hill	3,143 (38.96)
Terai	3,601 (44.63)
**Ethnic Group**	
Brahmin/Chhetri	3,305 (40.96)
Newar	326 (4.04)
Dalit	1,138 (14.11)
Janajati	2,588 (32.08)
Othe**r**	711 (8.81)
**Household Head**	1,659 (20.56)
**Education**	
None	4,069 (50.44)
Primary	1,513 (18.76)
Secondary	2,055 (25.47)
Higher	430 (5.33)
**Professional Occupation**	279 (3.46)
**Mean decision score (1–6)**	1.27 (0–2)
**Ratio of Boys/Girls**	1.19 (0.5–2)
**Total number of living children**	2.76 (2–4)
**Currently using modern method of family planning**	4,029 (49.94)

^#^(Currently married, non-pregnant women with at least 1 child)

### Household position

In unadjusted analysis, co-residing in a household with in-laws where there are no other sisters-in-law is associated with lower family planning use (Odds Ratio (OR) = 0.555, p<0.01) and co-residing in a household where the woman is the younger sister-in-law is associated with lower family planning use (OR = 0.415, p<0.01) compared to non-co-residing women (Table 2). After adjusting for other individual and household variables, co-residing in a household where the woman have no sisters-in-law (OR = 0.445, p<0.01), is the older sister-in-law (OR = 0.647, p<0.01) or younger sister-in-law (OR = 0.416, p<0.01) is significantly associated with lower family planning use. Being in an older age group, Hindu, in a higher wealth quintile, having a higher decision-making score, more children, and more boys compared to girls are all significantly associated with higher odds of family planning use. Being from an “other” ethnic group or being the head of the household is associated with lower odds of family planning use.

**Table 2:** Multi-level regression of the relationship between women’s position in the household and use of modern family planning (Odds Ratio, Standard Error).

	Use of modern family planning method unadjusted OR (SE)	Use of modern family planning method adjusted OR (SE)
**Position in the household (compared to not co-residing)**		
**Co-reside, no sisters-in-law**	0.555***	0.445***
	(0.469–0.657)	(0.367–0.539)
**Co-reside, older sister-in-law**	0.958	0.674***
	(0.815–1.127)	(0.562–0.808)
**Co-reside, younger sister-in-law**	0.415***	0.416***
	(0.333–0.518)	(0.323–0.536)
**Women’s Age (10 year age groups, compared to <20 years)**		
**20–29**		1.409
		(0.934–2.125)
**30–39**		2.553***
		(1.660–3.927)
**40+**		1.672**
		(1.031–2.712)
**Urban (vs. rural)**		1.176
		(0.945–1.464)
**Geographic Region (compared to mountain)**		
**Hill**		0.792*
		(0.604–1.038)
**Terai**		0.959
		(0.728–1.263)
**Ethic Group (compared to Brahmin/Chhetri)**		
**Newar**		1.406*
		(0.974–2.029)
**Dalit**		0.93
		(0.735–1.176)
**Janajati**		1.234*
		(0.996–1.528)
**Other**		0.729**
		(0.540–0.984)
**Head of Household**		0.195***
		(0.151–0.251)
**Education (compared to none)**		
**Primary**		0.965
		(0.790–1.178)
**Secondary**		0.908
		(0.739–1.115)
**Higher**		0.872
		(0.588–1.295)
**Occupation (Professional vs. Other)**		1.115
		(0.719–1.729)
**Hindu**		1.541***
		(1.150–2.066)
**Wealth Index (compared to poorest)**		
**Poorer**		1.443***
		(1.135–1.834)
**Middle**		1.855***
		(1.400–2.458)
**Richer**		2.036***
		(1.517–2.733)
**Richest**		2.552***
		(1.854–3.512)
**Decision score**		1.115***
		(1.054–1.179)
**Total number of living children**		1.083**
		(1.015–1.155)
**Ratio of Boys/Girls**		1.449***
		(1.337–1.572)
**Constant**	1.361***	0.238***
	(1.230–1.506)	(0.135–0.420)
**Number of Observations**	7,460	7,459

*p<-.01

**p<0.05

***p<0.01

### Food insecurity

Living in a moderately or severely food insecure household is associated with lower odds of family planning use (OR = 0.768, p<0.01 and OR = 0.690, p<0.01, respectively), compared to living in a non-food insecure household. After controlling for the individual and household variables, only living in a severely food insecure household is significantly associated with lower odds of family planning use (OR = 0.765, p<0.01). All of the same covariates are associated with higher or lower odds of family planning use as in the previous model (Table 3).

**Table 3:** Multi-level regression of the relationship between food insecurity, and use of modern family planning (Odds Ratio, Standard Error).

	Use of modern family planning method OR (SE) unadjusted	Use of modern family planning method OR (SE) adjusted
**Household Food Insecurity (FI) (compared to food secure)**		
**Mild FI**	0.925	1.005
	(0.762–1.123)	(0.819–1.233)
**Moderate FI**	0.768***	0.917
	(0.648–0.909)	(0.768–1.096)
**Severe FI**	0.690***	0.765***
	(0.573–0.831)	(0.625–0.937)
**Women’s Age (10 year age groups, compared to <20 years)**		
**20–29**		1.612**
		(1.086–2.394)
**30–39**		3.266***
		(2.162–4.934)
**40+**		2.208***
		(1.387–3.513)
**Urban (vs. rural)**		1.275**
		(1.026–1.584)
**Geographic Region (compared to mountain)**		
**Hill**		0.782*
		(0.597–1.023)
**Terai**		0.975
		(0.741–1.284)
**Ethic Group (compared to Brahmin/Chhetri)**		
**Newar**		1.346
		(0.930–1.948)
**Dalit**		0.959
		(0.756–1.217)
**Janajati**		1.183
		(0.951–1.471)
**Other**		0.700**
		(0.506–0.966)
**Head of Household**		0.243***
		(0.191–0.310)
**Education (compared to none)**		
**Primary**		0.92
		(0.752–1.125)
**Secondary**		0.830*
		(0.674–1.022)
**Higher**		0.75
		(0.510–1.103)
**Occupation (Professional vs. Other)**		1.168
		(0.765–1.782)
**Hindu**		1.475***
		(1.105–1.969)
**Wealth Index (compared to poorest)**		
**Poorer**		1.396***
		(1.100–1.773)
**Middle**		1.688***
		(1.264–2.255)
**Richer**		1.835***
		(1.355–2.484)
**Richest**		2.435***
		(1.751–3.385)
**Decision score**		1.119***
		(1.059–1.183)
**Total number of living children**		1.119***
		(1.051–1.191)
**Ratio of Boys/Girls**		1.459***
		(1.347–1.581)
**Constant**	1.345***	0.162***
	(1.191–1.518)	(0.0920–0.285)
**Number of Observations**	7,460	7,459

*p<-.01

**p<0.05

***p<0.01

### Interaction between household position and food insecurity

In the unadjusted model, living in a food secure, co-resident house with no sisters-in-law is associated with reduced odds of family planning use (OR = 0.635, p<0.01) and living in a food secure household as the younger sister-in-law is associated with lower odds of family planning use (OR = 0.387, p<0.01) compared to living in a food secure, non-co-resident household. Living in a food insecure, non-co-residing household (OR = 0.769, p<0.01), a food insecure, co-resident household with no sisters-in-law (OR = 0.320, p<0.01) and a food insecure household as the younger sister-in-law (OR = 0.347, p<0.01), are all associated with lower odds of family planning use, compared to living in a food secure, non-co-resident household. Elder sisters in food secure and insecure households do not have significantly lower odds of family planning use (Table 4).

**Table 4:** Multi-level regression of the relationship between food insecurity, women’s position in the household, and use of modern family planning (Odds Ratio, Standard Error).

	Use of modern family planning method OR (SE) unadjusted	Use of modern family planning method OR (SE) adjusted
**Interaction between Household Food Insecurity (FI) and Household Position (Baseline No FI and non-co-residing)** ^#^		
**No FI; co-reside no sisters**	0.635***	0.475***
	(0.520–0.774)	(0.379–0.593)
**No FI; co-reside, older sister-in-law**	0.925	0.641***
	(0.755–1.134)	(0.514–0.800)
**No FI; co-reside, younger sister-in-law**	0.387***	0.386***
	(0.284–0.528)	(0.277–0.539)
**FI; non-co-residing**	0.769***	0.845*
	(0.649–0.910)	(0.706–1.011)
**FI; co-reside no sisters**	0.320***	0.321***
	(0.241–0.424)	(0.235–0.439)
**FI; co-reside, older sister-in-law**	0.767*	0.608***
	(0.566–1.039)	(0.435–0.850)
**FI; co-reside, younger sister-in-law**	0.347***	0.393***
	(0.248–0.486)	(0.271–0.570)
**Women’s Age (10 year age groups, compared to <20 years)**		
**20–29**		1.378
		(0.912–2.082)
**30–39**		2.468***
		(1.603–3.799)
**40+**		1.612*
		(0.994–2.614)
**Urban (vs. rural)**		1.192
		(0.957–1.483)
**Geographic Region (compared to mountain)**		
**Hill**		0.813
		(0.620–1.065)
**Terai**		0.989
		(0.752–1.301)
**Ethic Group (compared to Brahmin/Chhetri)**		
**Newar**		1.404*
		(0.973–2.027)
**Dalit**		0.96
		(0.756–1.219)
**Janajati**		1.220*
		(0.985–1.512)
**Other**		0.733**
		(0.541–0.995)
**Head of Household**		0.192***
		(0.149–0.247)
**Education (compared to none)**		
**Primary**		0.958
		(0.782–1.174)
**Secondary**		0.885
		(0.721–1.086)
**Higher**		0.84
		(0.568–1.240)
**Occupation (Professional vs. Other)**		1.114
		(0.721–1.720)
**Hindu**		1.535***
		(1.147–2.055)
**Wealth Index (compared to poorest)**		
**Poorer**		1.405***
		(1.101–1.794)
**Middle**		1.762***
		(1.317–2.357)
**Richer**		1.883***
		(1.386–2.558)
**Richest**		2.321***
		(1.663–3.241)
**Decision score**		1.119***
		(1.057–1.184)
**Total number of living children**		1.084**
		(1.016–1.156)
**Ratio of Boys/Girls**		1.448***
		(1.336–1.570)
**Constant**	1.517***	0.271***
	(1.336–1.724)	(0.153–0.482)
**Number of Observations**	7,460	7,459

*p<-.01

**p<0.05

***p<0.01

^#^Bivariate of food insecurity was none/mild/moderate vs severe

After controlling for individual and household variables, all co-residing women have a significantly lower (p<0.05) odds of family planning use compared to non-co-residing, food secure women, with the exception of non-co-residing women in food insecure households (who have a lower odds only at the p<0.1 level). Women who are the younger sisters-in-law or co-resided with no sisters-in-law, had the lowest odds, in both food insecure and secure households. As before, being older, Hindu, higher on the wealth index, having a higher decision-making score, more children, and more boys compared to girls are all associated with higher odds of family planning use in this model. Also, being from an “other” ethnic group or the household head are significantly associated with lower odds of family planning use.

## Discussion

In this population-based study in Nepal, women’s position in the household is associated with lower odds of family planning use, with lower status women (younger sisters-in-law in co-resident households) and women with no sisters-in-law being least likely to be using family planning, even after controlling for other household and demographic variables. Women who are the older sister-in-law were less likely to use family planning after controlling for other factors, however, the odds were not as low as for younger-sisters-in-law or women with no sisters-in-law. While past research has highlighted the differences that may exist between co-residing and non-co-residing women, less research has explored differences by co-residence status [16]. One past study in India that did separate lower and higher status daughters-in-law within households found lower ranking daughters had children who were shorter than the children of higher ranking daughters [34]. Our findings add to these to suggest that not all co-resident women are the same and that understanding a women’s standing among the other women in her household could be important for understanding reproductive health outcomes and behaviors.

We also find that food insecurity is associated with lower odds of use of a modern family planning method, controlling for other household and individual level factors. These findings support past research in other countries that food insecurity is associated with poor health outcomes for women and children, and extend that work to include family planning [25, 26]. As discussed by Tsai et al. (2014) when looking at HIV and condom use in Nepal, the pathway from food insecurity to poor reproductive health outcomes is likely rooted in gender norms and inequality intersecting with food insecurity to put women at especially high risk of the marginalization and lack of economic security with which food insecurity is associated [28]. Past research in Uganda has found that women who are food insecure felt less empowered to negotiate for the use of family planning, a possible explanation for our findings in Nepal [35]. Given that the majority of households in Nepal experience some level of food insecurity (with 1 in 7 reporting severe food insecurity), reducing food insecurity could improve maternal and reproductive health indicators.

The interaction between food insecurity and household position suggests that younger sister-in-laws and co-residing women without sisters, are least likely to be using family planning in both food secure and food insecure households, even controlling for age, number of children and the gender of the children. This could suggest that lower status women, especially those in vulnerable households, are less able to access reproductive health services such as family planning. It appears that older sisters only have reduced odds of using family planning when they live in food insecure households. Our findings support past literature that a woman’s position in the household hierarchy is an important factor in understanding her health outcomes and behaviors [20]. Past research in Egypt described how when young married women moves in with their mothers-in-law, the daughters-in-law lose weight, while the mothers-in-law gain weight [36]. More data on the status of the mothers-in-law could help us understand if this phenomenon is occurring in Nepal as well. Having a higher decision-making score, an indicator of empowerment, is independently positively associated with family planning use after controlling for household position, suggesting that measurement of empowerment is complex, thus decision making power and household status should be assessed separately. Interestingly, being the household head, a measure often used for status or empowerment, is associated with lower odds of use in all models, suggesting that it might not be an appropriate measure for higher status/empowerment. It is also possible that given high levels of male out-migration in Nepal this variable is simply measuring lack of exposure to sex and therefore, lack of need of family planning. Given past research on geographic heterogeneity in both food security and women’s status, it was surprising that the variable for region of the country was not significant in any models [11, 23]. It is possible that other factors related to geography (poverty, ethnicity, religion) mask the impact of this measure alone.

This study has several important limitations. Many women in the “younger” sister category (Group 4) were dropped from the analysis because women who were pregnant or had not had any children were excluded. This reduced the sample size of this population and excluded women who were most likely at the lowest status in the household (had not had any children yet). Second, we are unable to assess causality or the direction of causality due to the cross-sectional nature of the survey data. Longitudinal research on the relationship between food insecurity and family planning use over time would help better understand the direction of this relationship. Furthermore, the survey measured food insecurity at the household level, not at the individual level, which makes it impossible to evaluate the different levels of food insecurity for different household members. We tried to account for this by including the variable for women’s status in the household. The variable that we used to measure household status only reflected the women in the household who were in the DHS sample, and there could have been other women in the household who were not in the survey but whose role in the household changed household dynamics around eating. Despite these limitations, this paper has several strengths. First, it adds nationally representative and more recent information to our understanding of factors associated with lower use of family planning in Nepal, a country where family planning use has not risen as much as anticipated. Additionally, it moves beyond simply exploring the impact of a single factor on family planning use, and instead looks at interactions, specifically how women’s individual status in the household interacts with a household level phenomenon (food insecurity) to compound risk.

This analysis suggests that women of the lowest status in food insecure, co-resident households appear to be especially at risk of not using modern family planning. It also suggests that not having any sisters-in-law could put women at risk of not being able to access services. These findings have important program and policy implications, suggesting that improving household level food insecurity can have important implications for women’s health and reproductive behaviors. It also suggests that focusing on the lowest status women in households could make the most impact for certain health behaviors and should be a priority. While improving overall food security should be the goal, policies or interventions that are able to improve women’s household status or improve the distribution of resources within the household, may have additional benefit.
